# Supplementary material for: Transcriptome Changes in Glioma Cells Cultivated under Conditions of Neurosphere Formation
Source: Cells. 2022 Oct 2;11(19):3106. doi: 10.3390/cells11193106 (PMC9563256; doi:10.3390/cells11193106)
Supplement: Supplementary file 1 [file cells-11-03106-s001.zip › Table S1.pdf]

Table S1. Sequences of primers used for the real time RT-PCR analysis of RNA.

**Primer ID**	**Nucleotide structure**
GAPDH_F	5′ - GAAGGTGAAGGTCGGAGT - 3′;
GAPDH_R	5′ - GAAGATGGTGATGGGATTTC - 3′;
SOX2_F	5′ - AACCCCAAGATGCACAACTC - 3′;
SOX2_R	5′ - GCTTAGCCTCGTCGATGAAC -3′;
ERRFI_F	5′ - GCTGCTCAGGAGATCAGAGTC - 3′;
ERRFI_R	5′ - GTAGGCCATGGTTATCGGGT - 3′;
TRIB2_F	5′ - GAGACTCCGAACTTGTCGCA -3′;
TRIB2_R	5′ - GAGCAGACAGGCAAAAGCAC - 3′;
NRP2_F	5′ - ACCTGGAGCATGACCCTTTG - 3′;
NRP2_R	5′ - GAGGATCCCCGTCGATGAAC - 3′;
NFKBIA_F	5′ - TGTCTACACTTAGCCTCTATC -3′;
NFKBIA_R	5′ - TCTGTGAACTCCGTGAACTC -3′;
CXCL1_F	5′ - TCTGGTCAGTTGGATTTGTCA -3′;
CXCL1_R	5′ - GCTGCCAGTGCTTGCAGACCCT -3′;
PDGFRA_F	5′ - TGTGGAGAATCTGCTGCCTG -3′;
PDGFRA_R	5′ - CCTCCCAGTCCTTCAGCTTG -3′;
ZEB1_F	5′ - CAATGATCAGCCTCAATCTGCA -3′;
ZEB1_R	5′ - CCATTGGTGGTTGATCCCA - 3′
