# Supplementary material for: Transcriptome Changes in Glioma Cells Cultivated under Conditions of Neurosphere Formation
Source: Cells. 2022 Oct 2;11(19):3106. doi: 10.3390/cells11193106 (PMC9563256; doi:10.3390/cells11193106)
Supplement: Supplementary file 1 [file cells-11-03106-s001.zip › Table S5.pdf]

Table S5. Validation of RNA-Seq results using real-time PCR (qRT-PCR). Eight randomly selected genes were analyzed by real-time PCR using RNA from independent replicas of adhesive and neurosphere glioma culture preparations. Represented the R-squared (R^2) linear regression values between relative expression obtained by DeSeq2 normalized gene/GAPDH RNA-seq values and qPCR genes levels normalized to the levels of GAPDH.

#	**Gene**	**Pearson R^2**
**1**	CXCL1	0.84
**2**	ERRFI1	0.93
**3**	NFKBIA	0.82
**4**	NRP2	0.81
**5**	PDGFRA	0.97
**6**	SOX2	0.72
**7**	TRIB2	0.68
**8**	ZEB1	0.60
